# Optimizing the heat transfer characteristics of MWCNTs and TiO_2_ water-based nanofluids through a novel designed pilot-scale setup

**DOI:** 10.1038/s41598-022-19196-3

**Published:** 2022-09-07

**Authors:** Reza Javadpour, Saeed Zeinali Heris, Yaghoub Mohammadfam, Seyed Borhan Mousavi

**Affiliations:** 1 Faculty of Chemical and Petroleum Engineering, University of Tabriz, Tabriz, Iran; 2 J. Mike Walker ’66 Mechanical Engineering Department, Texas A&M University, College Station, TX USA

**Keywords:** Carbon nanotubes and fullerenes, Nanoparticles, Chemical engineering, Energy storage

## Abstract

This study aimed to investigate the effect of titanium dioxide (TiO_2_) nano additives on the thermal performance of a pilot-scale cross-flow cooling tower. Moreover, it is a continuation of our previous study on the effect of using multi-walled carbon nanotubes (MWCNTs) nanofluid, and the results were compared with the results of TiO_2_ and previous work. An experimental design by response surface methodology (RSM) based on central composite design (CCD) with two factors (concentration and flow rate) was used to study the effectiveness of the setup, Merkel number, and the cooling range. The nanofluids were prepared by the two-step method. The stability tests were performed considering different surfactants such as Gum Arabic, Triton X-100, and sodium dodecyl sulfate, and Gum Arabic was determined as the optimal surfactant. The visual method, dynamic light scattering (DLS), and Zeta potential analyses were used to ensure the stability of the nanofluids and determine the size distribution of the nanoparticles in the nanofluids. The findings revealed that the heat transfer characteristics of the working fluid were improved with the addition of nanoparticles. Moreover, by comparing the effect of nanoparticles, it was found that MWCNTs could enhance the thermal features better than TiO_2_. The nanofluid containing 0.085 wt% of the MWCNTs improves the Merkel number, effectiveness, and cooling range by 28, 10.2, and 15.8%, respectively, whereas these values for TiO_2_ containing nanofluids are 5, 4.1, and 7.4%, respectively. MWCNTs nanofluid with a concentration of 0.069 wt% and a flow rate of 2.092 kg/min was proposed for optimal system setup. Under these conditions, the cooling range, effectiveness, and Merkel number were about 23.5, 55.75%, and 0.64, respectively.

## Introduction

Nanofluid is defined as a stable suspension of low nanoparticles content in the range of 1–100 nm in the base fluids such as oil, water, and ethylene glycol^1^. Recently, considerable studies have been dedicated to studying the heat transfer enhancement utilizing nanofluids in different applications such as cooling and refrigeration systems, process engineering, combustion engine, HVAC (heating, ventilation, and air-conditioning), power generation, and mechanical tools, and many others^2–4^. Heat transfer and thermophysical characteristics such as viscosity^5^, flash point, thermal conductivity, pour point, heat and mass transfer coefficient, and cooling rate can be enhanced utilizing nanofluids^6^. There is a broad type of nano additives that have been used in the preparation of nanofluids such as metal and metal oxides^7,8^, carbon-based nanomaterials^9,10^; however, although they have remarkable features like small size, large surface area, and excellent heat capacity, they tend to be agglomerated, especially at high concentrations. Preparing a stable nanofluid is still a challenge, many solutions address this commonly associated problem with nanoparticles, viz. surface modification methods^11^, ultrasonic agitation^12^, utilizing surfactants^13^, and pH treatment^14^. TiO_2_ nanoparticles have been widely used among different commonly utilized nano additives due to their distinctive properties. These include excellent colloidal and chemical stability, environmentally friendly^15^, heat transfer enhancement capability^16^, and friction-reduction behavior.

In assessing the heat transfer characteristics of a cooling system, MWCNTs/nanofluids have shown a significant enhancement in the measured thermophysical properties such as thermal conductivity since CNTs possess almost 5 times higher value than other conventional materials^17^. Consequently, the higher thermal conductivity of MWCNTs/nanofluid ensures a better heat transfer rate in the applied system^18^.

Among traditionally used cooling systems, the cooling tower has been used in diverse applications where waste heat is needed to be eliminated from the system. The procedure principle of the cooling tower using water involves direct contact between two fluid streams of water and unsaturated air due to the difference in vapor concentration between water and gas phases. Accordingly, water vaporizes and cools down while the air wets and becomes warmer. The efficiency of a cooling tower depends on many parameters, including fluid flow rate, inlet conditions of the used fluid, and the utilized elements in the system^19^. Cooling towers are classified into three flow patterns: cross-flow, parallel-flow, and counter-flow^20^. In terms of using a fan, cooling systems are divided into natural draft and mechanical draft cooling towers^21^.

Ayoub et al.^22^ investigated the impact of weather variables on the performance of a wet cooling tower. Their findings revealed that even a minor temperature increase relative to the cooling tower's design temperature dramatically affects its effectiveness. Li et al.^23^ presented a novel method to improve cooling tower performance. They found that optimizing the water mass flow in air heat exchangers could significantly reduce the damaging effects of crosswind on cooling tower performance. Lyu et al.^24^ operated a 3-D numerical model to analyze the influence of various fill arrangement designs on the cooling tower performance. They found that the non-uniform arrangement could enhance cooling tower performance in both crosswind and windless states. Imani Mofrad et al.^25^ evaluated the effect of 6 various types of filled beds on the cooling tower performance by using a ZnO nanofluid. They observed that the reticular metal bed showed the best performance. In another study, Imani Mofrad et al.^26^ examined the impact of different nanoparticles such as graphene, ZnO, Al_2_O_3_, and SiO_2_ on the cooling tower performance. The results confirmed that graphene nanoparticles provided the most remarkable improvement in tower performance. Amini et al.^27^ prepared Al_2_O_3_ and CuO water-based nanofluids at diverse concentrations and assessed their effect on the mechanical draft cooling tower performance considering different inlet temperatures. They found that the prepared nanofluids improved the cooling tower performance, and this improvement depended on the type, concentration, and inlet temperature of nanofluid. Javadpour et al.^28^ scrutinized the effect of operating parameters on tower performance in a cross-flow cooling tower utilizing MWCNTs nanofluid as the working fluid. The outcomes exhibited that nanofluids had a more substantial influence on tower performance at lower flow rates. Furthermore, nanofluids containing 0.085 wt% nanoparticles work best, with a 15.8 percent improvement in cooling range and a 10.2 percent increase in ineffectiveness. Rahmati^29^ conducted a study to experimentally examine the effect of ZnO nanofluid on the thermal performance of a mechanical draft wet cooling tower considering different concentrations and packing types. It was reported that the cooling efficiency could be improved with the addition of ZnO nanoparticles in the water. Furthermore, it was highlighted that better performance was observed with the increase of packing layers. Alklaibi et al.^30^ experimentally assessed the usages of MWCNTs/water-based nanofluid as a cooling agent at diverse volumetric concentrations. Their findings showed that the prepared nanofluids' heat transfer and thermophysical properties were enhanced by adding the MWCNTs as additives. The maximum thermal performance factor and effectiveness rate were observed for 0.3 vol% MWCNTs nanofluid with a value of 1.12 and 13.21% at a 7 lit/min flow rate.

According to the conducted literature review, most research studies in cooling systems have focused on improving the cooling towers' performance considering different affecting factors such as environmental conditions, physical components, and operating conditions. Nonetheless, the effect of using nanoparticles in the preparation of the working fluid in a system has not been well-understood. To the best of our knowledge, in terms of flow pattern, most studies have focused on counter-flow cooling towers, while none of the studies considered cross-flow towers using TiO_2_ nanofluids. The complexity and difference in solving the governing equations (which must be solved by numerical methods) related to the Merkel number (transfer characteristic) of cross-flow cooling towers in terms of the temperature gradient in the horizontal and vertical directions could be the main reason. For this reason, the Merkel number was not considered in our previous research; hence it is calculated and compared in this study. On the other hand, the Merkel number is the most important factor in evaluating cooling towers' performance. As a dimensionless number, it is a good measure for comparing the thermal performance of cooling towers. Accordingly, to compensate for the gap in the previous study, it was calculated and compared for both nanofluids in this study. It is worth stating that different affecting factors the cooling tower performance such as type of nanoparticle, nanoparticles concentration, and fluid flow rate were comprehensively scrutinized in this study.

In this examination, two different water-based nanofluids using MWCNTs and TiO_2_ nanoparticles were prepared. The effect of nanofluid flow rate and concentrations on the cooling tower performance was evaluated using an experimental design by response surface methodology (RSM) based on the central composite design (CCD). The effectiveness, Merkel number, and cooling range were also measured. Furthermore, the ideal and economic optimization for various parameters were presented. Meanwhile, the authors' previous halfway study on the effects of using nanofluid made of MWCNTs was continued and completed in this study, and the previous outcomes were compared with the concurrent results of TiO_2_.

## Material and method

### Experimental setup

**Figure 1:**
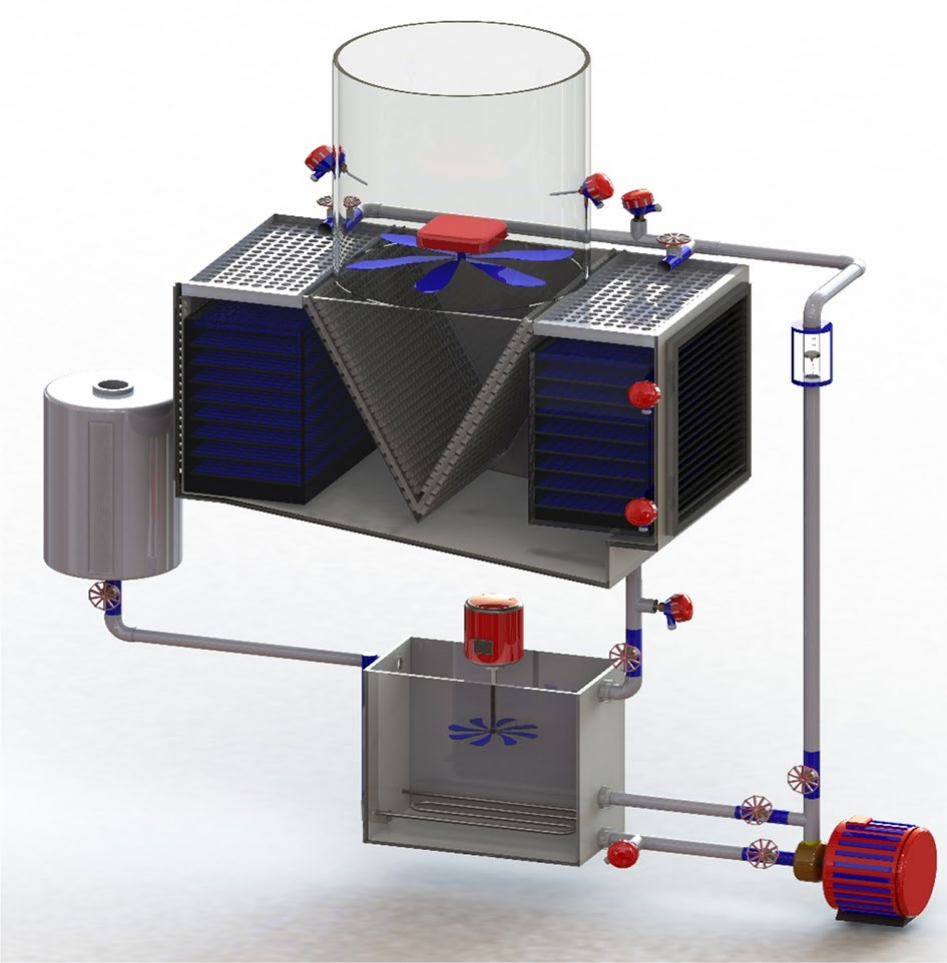
Schematic of the designed setup.

**Figure 2:**
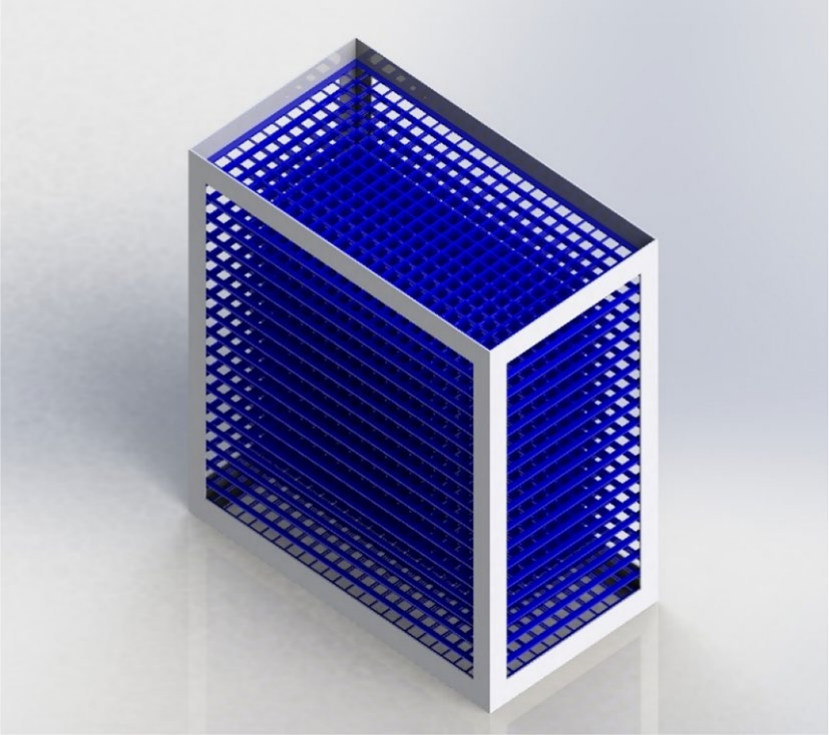
Schematic of the used filled bed.

Figure 1 presents the schematic of the experimental system designed in Solid works 2021 SP5. The tower's main body consists of a polycarbonate-made square cross-section with dimensions of 0.5 × 0.5 × 1 m. The heating part of the experimental system, which is used to increase the temperature of the inlet working fluid, contains a fluid height indicator, mixer, tank, and element. The most important part of the cooling tower is the filled bed, where the main processes are performed to reduce the temperature of the working fluid. The schematic of the used filled bed is exhibited in Fig. 2. After heating, the operating fluid is transferred to the top of the tower employing a centrifugal pump and then spread on the filled bed utilizing a designed distribution system for the uniform distribution of fluid inside the tower. A make-up water tank has been used to replace the working fluid that evaporates during the process. In addition, two aluminum-made droplet eliminators have been used to prohibit working fluid droplets from escaping. To measure the elements' temperature and flow rate of the working fluid, PT-100 resistance temperature detectors (RTDs), and rotameter have been installed.

### Preparation and characterization of nanofluid

**Figure 3:**
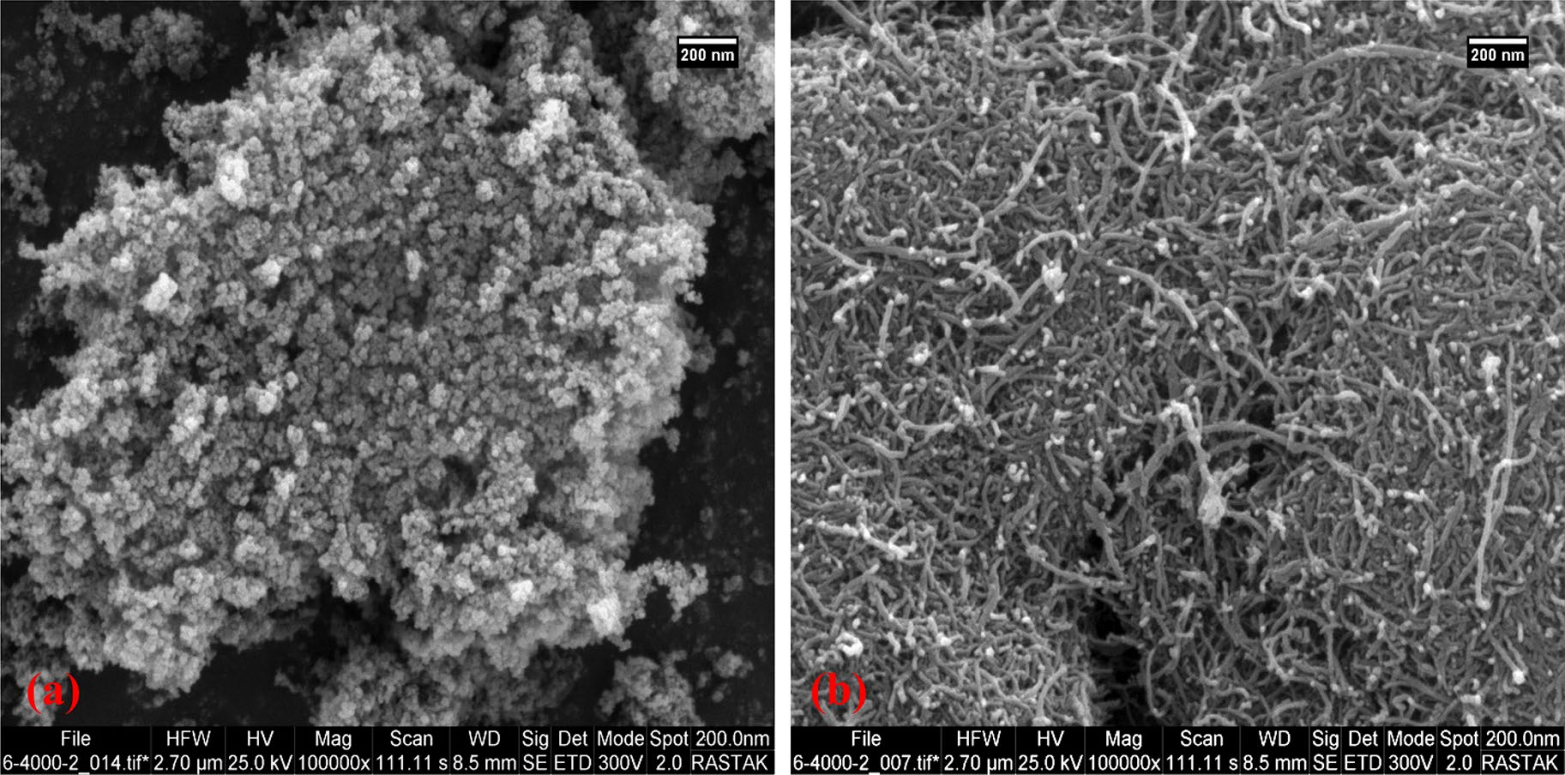
SEM images of (**a**) TiO_2_, (**b**) MWCNTs.

MWCNTs and TiO_2_ nanoparticles were purchased from VCN and Sigma-Aldrich, respectively. The properties of these nanomaterials and the SEM images are presented in Table 1 and Fig. 3, respectively. The gum Arabic, Triton-X-100, and sodium dodecyl sulfate as the surfactants were purchased from Merck, Germany.

**Table 1:** Characteristics of nanomaterials.

Nanomaterial	TiO_2_	MWCNTs
Chemistry formula	TiO_2_	C
Color	White	Black
Purity	99.8	> 95%
Average diameter (nm)	15	15
Morphology	Spherical	Multi-wall hollow tubes
Special area (m^2^/g)	60–80	> 200
Special heat capacity (J/g C)	0.68	0.78
Density (g/cm^3^)	4.23	2.1

**Figure 4:**
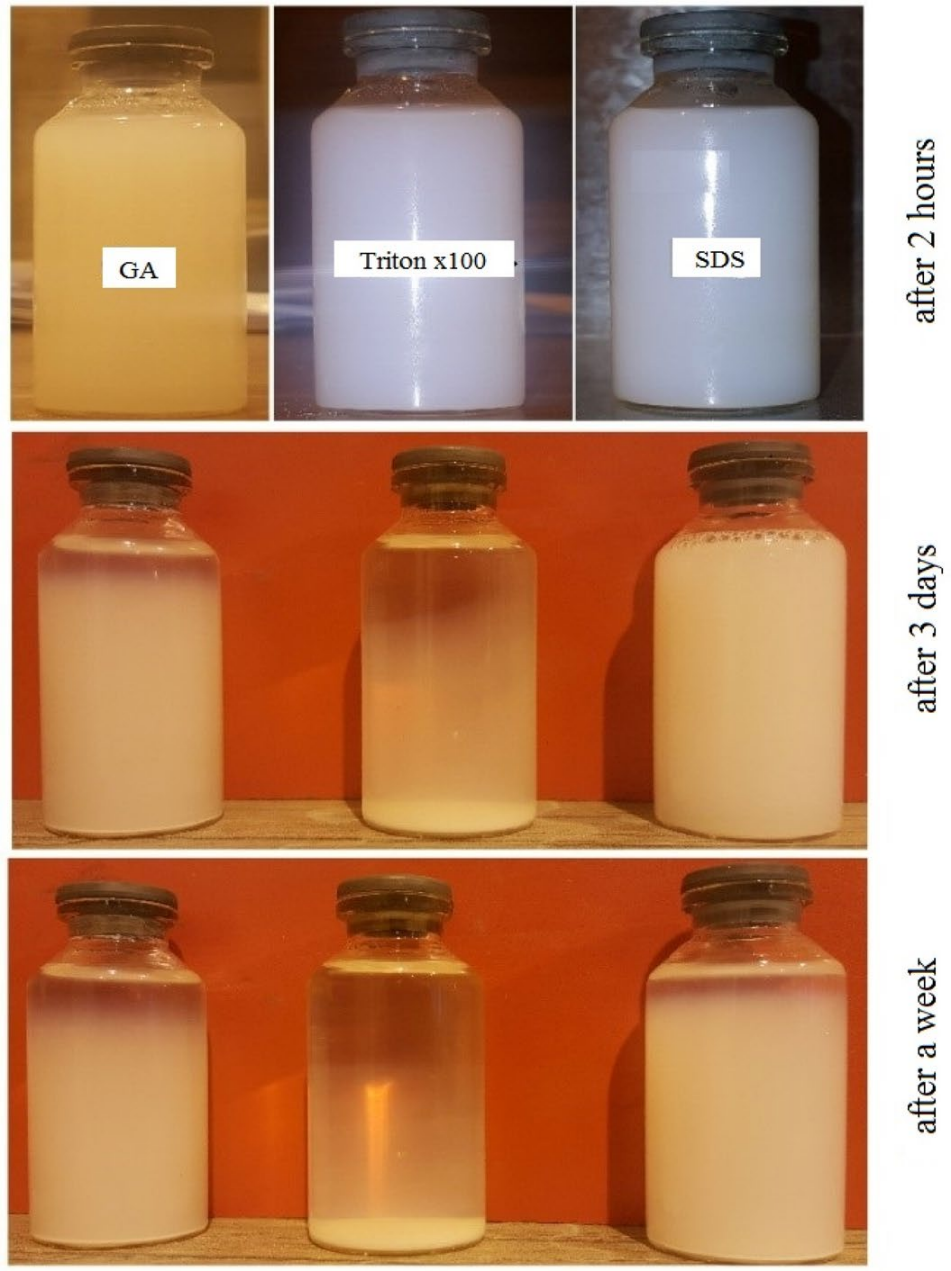
The Impact of different surfactants on nanofluid stability.

**Figure 5:**
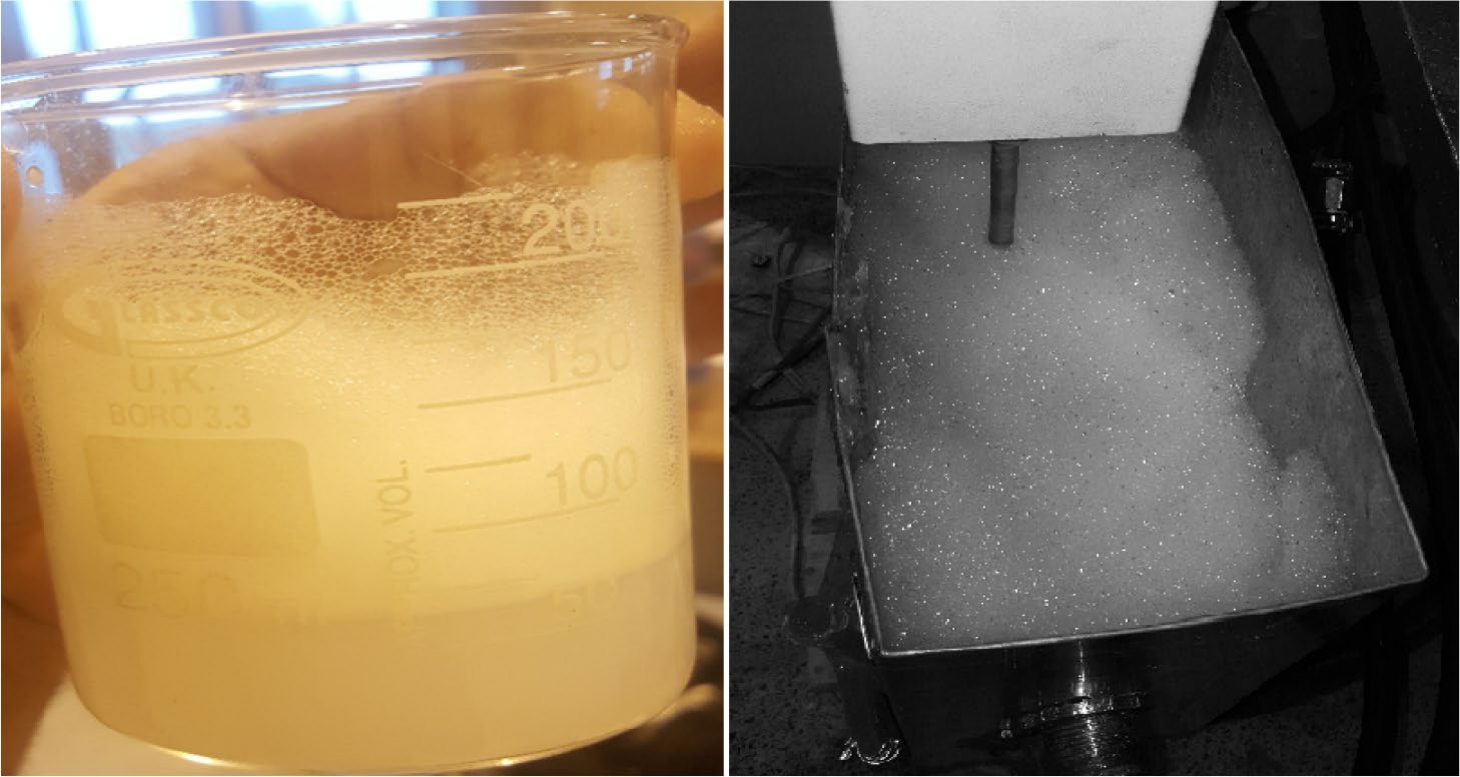
Formation of foam when using sodium dodecyl sulfate as a surfactant.

The two-step method was employed to prepare the nanofluids. The surfactant was weighed in a 1: 1 ratio of nanomaterials and mixed with 10 L of water utilizing a mechanical stirrer at 1300 rpm for 30 min. Then, MWCNTs and TiO_2_ nanoparticles were added at different concentrations, 0.015, 0.005, 0.085, and 0.1 wt%, to the prepared solution. The prepared nanofluid was exposed to ultrasonic waves for 4 h in an ultrasonic bath after stirring for 3 h using the mechanical stirrer at 1300 rpm. To select the most suitable surfactant among three different surfactants namely Gum Arabic, Triton X-100, and sodium dodecyl sulfate (SDS), the 0.1 wt% TiO_2_ nanofluid was prepared utilizing each surfactant. A qualitative stability test was conducted to investigate the effect of each surfactant. Figure 4 shows the result of conducted stability test of TiO_2_ nanofluids after 2 h, 3 days, and one week. It was observed that the stability of the nanofluid using sodium dodecyl sulfate was better than the other two surfactants. Although the nanofluid containing SDS had better colloidal stability among other considered surfactants, the Gum Arabic was selected as the appropriate surfactant since using SDS led to the formation of foam at the surface of the nanofluids, which is not suitable for the cooling tower system (Fig. 5). The stability analyses of MWCNTs nanofluids were presented in our previous work^28^.

**Figure 6:**
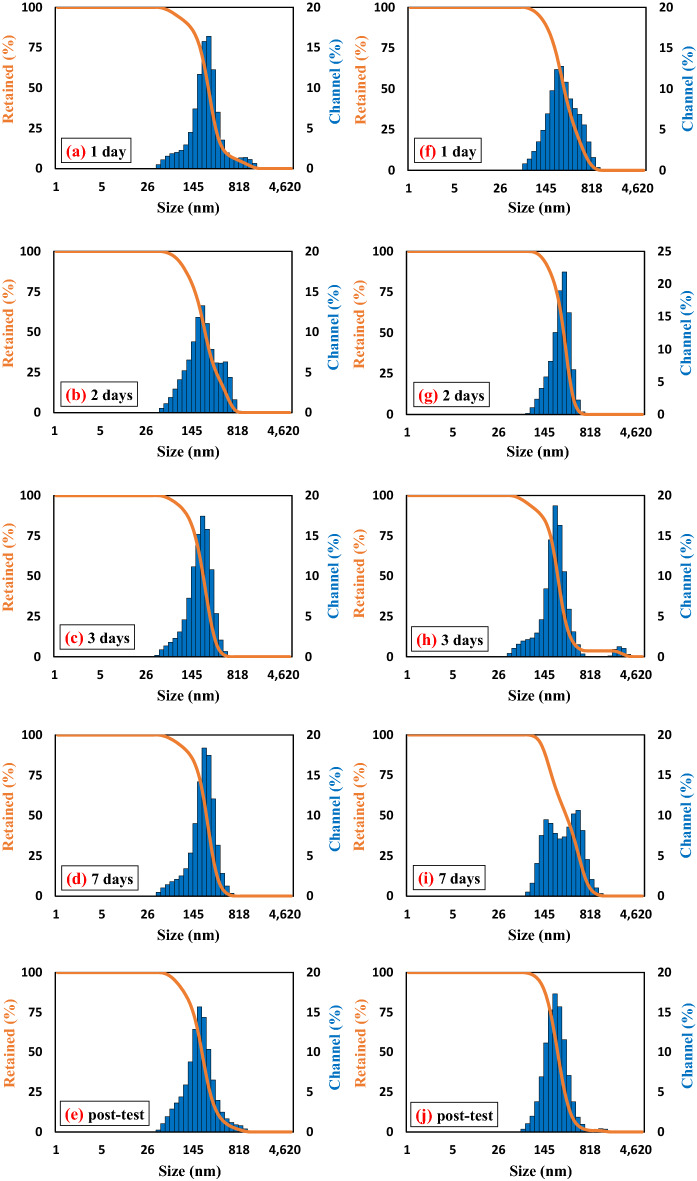
DLS data analysis for the nanofluids post-test and after several days: (**a**–**e**) MWCNTs, and (**f**–**j**) TiO_2_.

Dynamic light scattering (DLS) analysis was used to assess the size distribution of nanoparticles in the nanofluids and the change in the size of nanoparticle aggregations at 25 °C as a time function. The most concentrated sample of MWCNT and TiO_2_ nanofluids (0.1 wt%) was selected as the sample most prone to instability. The nanofluids were analyzed by the DLS method at time intervals of one day, two days, three days, and seven days after the nanofluids were prepared; the remaining nanofluid after the test was also evaluated and compared for the final analysis. The obtained size distributions are depicted in Fig. 6. It can be derived from the figures that the change in the size distribution of the nanoparticles in the MWCNT nanofluid was not very significant in contrast to the TiO_2_ nanofluid. Also, comparing the size distribution of the nanoparticles in the suspension before and after the experiment indicated that the stability of the nanofluid was maintained during the process. For the TiO_2_ nanofluid, there were no significant changes in the particle size after one day and two days after the nanofluid preparation and after performing the test, which is proof of the stability of the nanofluid during the process. Also, it showed reasonable stability at least two days after the nanofluid preparation. However, after three days of preparation of the TiO_2_ nanofluid, it was observed that the nanoparticles in Fig. 6h formed another small peak with a size of about 2500, indicating the beginning of aggregation of the nanoparticles. After seven days, the overall distribution of particles in the TiO_2_ nanofluid showed that the total size of nanoparticles increased. Thus, the DLS analysis confirmed that nanofluids made of MWCNTs with a particle distribution of about 220 nm and TiO_2_ with a particle distribution of about 270 nm were stable for at least seven and two days after preparation and immediately after testing, respectively, and that the results obtained from them can be relied upon.

Table 2 illustrates the average nanoparticle size distribution and zeta potential of MWCNT and TiO_2_ nanofluids at their natural pH according to Fig. 6. The average nanoparticle size distribution also showed that the particle size distribution for the MWCNT nanofluid hardly changed over time, while the average particle size for TiO_2_ gradually over time.

**Table 2:** Average particle size distribution and zeta potential of nanofluids.

Time (day)	MWCNTs			TiO_2_		
	Label in Fig. 6	Average particle size distributions (nm)	Zeta potential (mV)	Label in Fig. 6	Average particle size distributions (nm)	Zeta potential (mV)
1	a	219	45.1	f	270.1	41.9
2	b	223.9	41.6	g	281.9	41.6
3	c	209	43.2	h	306	39.5
7	d	222.4	42.7	i	340.5	23.1
After the test	e	211.9	44.4	j	254	40.8

The stabilization theory states that when the zeta potential is high (positive or negative), the electrostatic repulsions between particles increase, resulting in good suspension stability. Since the contact is opposite, particles with a high surface charge do not agglomerate. The generally accepted zeta potential values were summarized by Ghadimiet et al.^31^. The zeta potential is commonly used to index the extent of electrostatic interaction between colloidal particles. It can therefore be considered a measure of the colloidal stability of the solution^32^. The zeta potential results for MWCNT nanofluid confirmed an average value of about 43 for all time intervals, indicating reasonable stability of all suspensions. For the TiO_2_ nanofluid, the zeta potential value of about 41 showed that the nanofluids had good stability after the test and on the first and second days. For the third day, the zeta potential was inferred to be moderately stable (zeta potential of 39.5). However, as shown in Figs. 4 and 6, signs of instability gradually appeared after three days of nanofluid preparation. In summary, it is worth mentioning that MWCNT and TiO_2_ nanofluids were stable for at least seven and two days after preparation, respectively, and the results of the experiments are related to their stable state.

To ensure that the concentration (weight percent) of nanoparticles in the nanofluid remained constant after the experiment, the density of the nanofluids at all four concentrations prepared was measured and compared before and after the experiment (Table 3). Since there was evaporation in the system and was replaced by water, the density of the nanoparticles did not change significantly before and after the test cycle. To illustrate, at lower concentrations, the density results before and after the experiment were the same, and at two higher concentrations, the density of the nanofluids after the experiment was slightly lower than before the experiment. The reason is probably that a small amount of the nanofluids is trapped in the dead zones of the filled bed or water distribution system and is replaced by pure water. From the results that the density of the nanofluids remained approximately constant before and after the experiment, it can be concluded that the weight of nanoparticles per unit volume of fluid remained constant, indicating a constant total concentration of circulating fluid during the experiment.

**Table 3:** The density of nanofluid samples before and after the experiment.

	0.015 wt%		0.05 wt%		0.085 wt%		0.1 wt%	
	Befor	After	Befor	After	Befor	After	Befor	After
MWCNTs (g/cm^3^)	0.99620	0.99620	0.99660	0.99660	0.99690	0.9968	0.99720	0.9971
TiO_2_ (g/cm^3^)	0.9965	0.9965	0.9976	0.9976	0.9987	0.9986	0.9992	0.9990

## Formulation

This section provides the equations of some crucial parameters such as cooling range, efficiency, Merkel number, and evaporation rate to specify the cooling tower performance.

The cooling range, which is described as the difference between the temperature of the inlet hotfluid (${T}_{W,i}$) and the outlet ld fluid (${T}_{W,O}$), is obtained by the following equation^33^:

1 $$T_{R} = T_{w,i} - T_{w,o} .$$

The effectiveness of the CFCT (ɛ), the ratio of the temperature difference between cold and hot fluid to the maximum possible temperature difference, is obtained through Eq. (2)^34^.

2 $$\varepsilon = \frac{{T_{w,i} - T_{w,o} }}{{T_{w,i} - T_{a,wet,o} }}$$

where ${T}_{a,wet,i}$ is wet bubble inlet air temperature, ${T}_{W,O}$*,* is outlet fluid temperature, and ${T}_{W,i}$ is inlet fluid temperature.

The Merkel number, a transfer characteristic for evaluating and comparing the thermal performance of fills, is defined as follows^35^:

3 $$Me = \frac{{h_{d} a_{fi} A_{fr} L_{fi} }}{{m_{w} }} = \frac{{h_{d} a_{fi} L_{fi} }}{{G_{w} }} = \mathop \int \limits_{{T_{wo} }}^{{T_{wi} }} \frac{{C_{pw} dT_{w} }}{{\left( {I_{masw} - I_{ma} } \right)}}$$

where ${h}_{d}$, ${a}_{fi}$, ${A}_{fr}$, ${L}_{fi}$, ${m}_{w}$, ${G}_{w}$, ${C}_{pw}$, ${T}_{w}$, ${I}_{masw}$, and ${I}_{ma}$ are the mass transfer coefficient (m/s), the interfacial surface area between air and water per unit volume of fill zone(m^-1^), the frontal area of fill perpendicular to the airflow direction (m^2^), the fill length (m), the mass flow rate of water (kg/s), the mass velocity of water (kg/m ^2^.s^1^), the specific heat at constant pressure (J/kg.K), temperature (°C), the specific enthalpy of saturated air (per kg dry air) (J/kg), the specific enthalpy of the air-vapor mixture (per kg dry air) (J/kg), respectively.

The Merkel number is considered as a function of the water mass flow rate $({m}_{w})$, the minimum evaporative capacity rate ${(C}_{ min})$, and the number of transferred (heat) units (*NTU*) and is calculated using the *ɛ-NTU* method, as follows^35^:

4 $$Me=\frac{NTU {C}_{ min}}{{m}_{w}}$$

To obtain $NTU$ and ${C}_{ min}$, the system of equations must be solved simultaneously using a iteration method. This system of equations is given below^36^.

5 $$\varepsilon ={\left[\frac{1}{1-\mathrm{exp}(-NTU)}+\frac{C}{1-\mathrm{exp}(-C.NTU)}-\frac{1}{NTU}\right]}^{-1}$$

6 $$\varepsilon =\frac{Q}{{Q}_{max}}$$

7 $$Q={m}_{a}\left({I}_{mao}-{I}_{mai}\right)$$

8 $${Q}_{max}={C}_{min}\left({I}_{maswi}-\lambda -{I}_{mai}\right)$$

9 $$\lambda =\frac{\left({I}_{maswo}+{I}_{maswi}-2{I}_{masw}\right)}{4}$$

10 $${C}_{min}=min\left({m}_{w}{C}_{pw}/\frac{{dI}_{masw}}{{dT}_{w}},{m}_{a}\right)$$

11 $${C}_{max}=max\left({m}_{w}{C}_{pw}/\frac{{dI}_{masw}}{{dT}_{w}},{m}_{a}\right)$$

12 $$\frac{{dI}_{masw}}{{dT}_{w}}=\frac{{I}_{masw}-{I}_{maswo}}{{T}_{w,i}-{T}_{w,o}}$$

13 $$C=\frac{{C}_{min}}{{C}_{max}}$$

The following equation can be utilized to specify the evaporation rate^37^:

14 $${\text{Evaporation rate}} = K \times \left( {X_{o} - X_{i} } \right)$$

where $K$, *X*_*o*_, and *X*_*i*_ represent the inlet air flow rate, the specific humidity of the air at the outlet, and inlet of the tower, respectively.

When nanofluid is used instead of water as the working fluid, Eqs. (15) and (16) can be used to calculate the specific heat of the nanofluid^38^.

15 $${({C}_{P})}_{nf}=\left(1-\varphi \right){\left({C}_{P}\right)}_{bf}+\varphi {({C}_{P})}_{p}$$

16 $${({\rho C}_{P})}_{nf}=\left(1-\varphi \right){\left({\rho C}_{P}\right)}_{bf}+\varphi {({\rho C}_{P})}_{p}$$

where ${({C}_{P})}_{nf}$, ${\left({C}_{P}\right)}_{bf}$ and ${({C}_{P})}_{p}$, and $\varphi$ are the specific heat of the nanofluid, base fluid, and nanoparticles, and the volume concentration of nanofluids, respectively.

The methods presented by Holman^39^ and Sadri^40^ were used to calculate the uncertainty of the measured parameters. Table 4 shows the maximum error of the measured quantities.

17 $$U_{M} = \pm \left\{ {\left( {\frac{{y_{1} }}{M}\frac{{\partial M}}{{\partial y_{1} }}u_{{y_{1} }} } \right)^{2} + \left( {\frac{{y_{2} }}{M}\frac{{\partial M}}{{\partial y_{2} }}u_{{y_{2} }} } \right)^{2} + \ldots + ~\left( {\frac{{y_{n} }}{M}\frac{{\partial M}}{{\partial y_{n} }}u_{{y_{n} }} } \right)^{2} } \right\}^{{{\raise0.7ex\hbox{$1$} \!\mathord{\left/ {\vphantom {1 2}}\right.\kern-\nulldelimiterspace} \!\lower0.7ex\hbox{$2$}}}}$$

where ${y}_{i}$, ${{u}_{y}}_{i}$, ${U}_{M}$ are the measurable parameter, the measured error, and the maximum error of parameter $M$.

**Table 4:** The maximum inaccuracy in the obtained characteristics.

Characteristics	Maximum inaccuracy (%)
Effectiveness of the cooling tower (ɛ)	0.9
Cooling range (range)	0.4
The flow rate of the operating fluid	3
Evaporation rate (E)	0.4
The temperature (T, °C)	0.05
Mass velocity (G)	0.3
The mass of nanoparticle (m_np_)	0.5
The particle weight fraction (wt%)	0.5

## Experimental design and analysis

The goal of optimization is to find the best acceptable solution given the limitations and needs of the problem. Experimental design is a set of practical statistical methods for modeling and analyzing problems in which several variables affect the response level. For analyzing experiments, after determining the affecting variables of the process, it is vital to optimizing the influential variables to achieve the best and most appropriate response. One of the most critical advantages of the design of experiments (DOE) is determining the optimal conditions for the process. One of the most suitable optimization methods is RSM. RSM is a set of mathematical and statistical techniques used to develop experimental models. In such designs, the goal is to optimize the response (output variable) affected by several independent variables (input variables)^41^. In this work, a CCD-based experimental design via the RSM method was used to optimize circulating fluid flow rate and nanoparticles' weight percentage on the cooling tower performance. For this purpose, the Design-Expert version, 11.0.3.0, was used. Also, 5 levels were considered for each parameter based on the software default. The values of these factors are presented in Table 5. The characteristics of the proposed models have been described by a series of factors, such as coefficient of determination (R^2^), Fisher variation ratio (F-value), and adjusted coefficient of determination (Adj-R^2^).

**Table 5:** The levels of factors.

Levels	A: weight percentage of nanoparticles (wt%)	B: flow rate of operating fluid (kg/min)
$-$ 1.414	0	2
$-$ 1	0.015	2.6
0	0.05	4
1	0.085	5.5
1.414	0.1	6

## Results and discussion

### Changes in cooling range as a function of concentration and flow rate

Experiments have been performed under relatively constant environmental conditions using different operating fluids (distilled water, MWCNTs, and TiO_2_ nanofluids) and five levels of the RSM method. During the experiments, the flow rate of passing air and operating fluid was constant at 7.97 kg/min and 4 kg/min, respectively. After reaching the steady-state, the inlet hot water and outlet cold water temperatures were recorded, and the cooling range was calculated.

The design of experiment table for MWCNTs and TiO_2_ nanofluids by the CCD-based RSM method is presented in Table 6. The experimental design points of the response procedure were used in the factorial method. It means, instead of conducting 13 experiments for each nanofluid,29 experiments were performed, and the results were analyzed in the Historical Data section of the software. The total number of tests was 58, 29 for each nanofluid.

**Table 6:** The design of experiment table for MWCNTs and TiO_2_ nanofluids by the CCD-based RSM method.

Run	Factor 1	Factor 2	Response 1		Response 2		Response 3	
	A: concentration (wt%)	B: flow Rate (kg/min)	Range (°C)		Effectiveness (%)		*Me*	
			MWCNTs	TiO_2_	MWCNTs	TiO_2_	MWCNTs	TiO_2_
1	0	2	20.6	20.6	51.37	51.37	0.55	0.55
2	0	2.6	17.2	16.8	46.73	45.9	0.34	0.34
3	0	4	12.7	12.7	38.95	38.96	0.23	0.23
4	0	5.5	9.9	9.9	32.5	33.9	0.18	0.18
5	0	6	8.8	8.8	31.14	29.33	0.16	0.16
6	0.015	2	21.4	21	50.96	52.11	0.56	0.56
7	0.015	2.6	17.9	17.9	46.77	46.68	0.35	0.36
8	0.015	4	13.2	12.9	39.52	39.21	0.23	0.24
9	0.015	5.5	10.2	9.7	33.99	33.56	0.19	0.18
10	0.015	6	9.2	8.9	32.94	29.77	0.17	0.16
11	0.05	2	22.7	22.1	53.96	52.12	0.63	0.56
12	0.05	2.6	18.7	17.7	49.71	48.23	0.39	0.37
13	0.05	4	13.4	13.2	40.97	39.29	0.25	0.25
14	0.05	5.5	10.2	9.9	34.87	34.02	0.19	0.18
15	0.05	6	9.1	9.1	33.94	30.23	0.17	0.16
16	0.05	4	13.3	13.3	40.8	39.58	0.24	0.25
17	0.05	4	13.4	13.4	40.85	39.88	0.25	0.24
18	0.05	4	13.5	13.8	41.03	40.83	0.25	0.26
19	0.05	4	13.4	13	40.98	39.76	0.25	0.25
20	0.085	2	25.2	21.4	56.47	51.57	0.80	0.57
21	0.085	2.6	20.7	19.4	51.53	47.55	0.43	0.37
22	0.085	4	14.3	13.6	42.09	40.36	0.27	0.25
23	0.085	5.5	11	10.3	34.67	34.33	0.20	0.18
24	0.085	6	9.7	9.3	32.84	32.07	0.17	0.16
25	0.1	2	24.7	21.5	52.89	50.83	0.63	0.56
26	0.1	2.6	19.9	18.5	48.06	46.37	0.39	0.37
27	0.1	4	13.7	13.1	39.71	38.99	0.25	0.24
28	0.1	5.5	10.3	10.1	32.8	33.01	0.19	0.17
29	0.1	6	9.3	9.1	31.74	30.95	0.17	0.15

The analysis of variance (ANOVA) table for cooling range data of the tower using TiO_2_ nanofluid is given in Table 7. According to the software definition, terms with a P-value $>$ 0.1 are not significant and have little effect on the final equation and responses. Therefore, it is better to remove them from the final equation to increase the model's validity. All terms have a P-Value $<$ 0.1 and are not excluded from the final equation. The P-value of the Lack of Fit term is more than 0.05 and is not significant. The Lack of Fit F-value of 1.92 indicates that the Lack of Fit is insignificant compared to the pure error. A "Lack of Fit F-value" of this magnitude has a 27.84 percent chance of occurring due to noise.

**Table 7:** Cooling range data of TiO_2_ nanofluid from ANOVA.

Source	Sum of squares	df	Mean Square	F-value	*p*-value
Model	561.48	5	112.30	724.96	< 0.0001 Significant
A-concentration	2.46	1	2.46	15.88	0.0006
B-Flow Rate	545.28	1	545.28	3520.26	< 0.0001
AB	0.4943	1	0.4943	3.19	0.0872
A^2^	0.6303	1	0.6303	4.07	0.0555
B^2^	19.00	1	19.00	122.66	< 0.0001
Residual	3.56	23	0.1549	_	_
Lack of Fit	3.21	19	0.1690	1.92	0.2784 Not significant
Pure Error	0.3520	4	0.0880	–	–
Cor Total	565.04	28	–	–	–

The software presented a quadratic equation as the model equation. Equations (18) and (19) demonstrate the actual and coded equations to predict the effect of TiO_2_ nanofluid flow rate and concentration on the cooling range.

18 $$\mathrm{Range}=32.34+32.57\mathrm{C}-7.02\mathrm{L}-2.33\mathrm{CL}-151.15{C}^{2}+0.52{L}^{2}$$

19 $$\mathrm{Range}=13.33+0.41\mathrm{A}-5.98\mathrm{B}-0.23\mathrm{AB}-0.38{A}^{2}+2.07$$

To evaluate the proposed model's validity, the model's descriptive statistics for the tower using TiO_2_ nanofluid are given in Table 8.

**Table 8:** The descriptive statistics of the proposed model for cooling range.

Statistical summary of the model	Statistical summary of data
${R}^{2}=$ 0.9937	Std. Dev.$=$ 0.3936
Adjusted ${R}^{2}=$ 0.9923	Mean $=$ 14.17
Predicted ${R}^{2}=$ 0.9901	C.V.%$=$ 2.78
Adeq Precision $=$ 71.3596	–

The coefficient of variation (C.V.) has a low value showing low data scatter. ${R}^{2}=$ 0.9937 demonstrates that the proposed model can describe 99.37% of the cooling range changes. The Adj-R^2^ reveals the conformity degree between the experimental data and the model by considering the degree of model freedom and the number of experiments, and Adj-R^2^ $=$ 0.9923 indicates a 99.23% correlation between the model and experimental data. The ability of the model to predict points outside the defined levels is also significant and has a value of 99.01%. The difference between Pred-R^2^ and Adj-R^2^ is insignificant (based on the software default, Pred-R^2^ and Adj-R^2^ should not have more than 0.2 differences). Adeq Precision also has a significant value of 71.3596, which implies the favorable conditions of the model for use in industry. To use the predicted model for industrial purposes, the Adeq Precision must have a value higher than 4.

**Figure 7:**
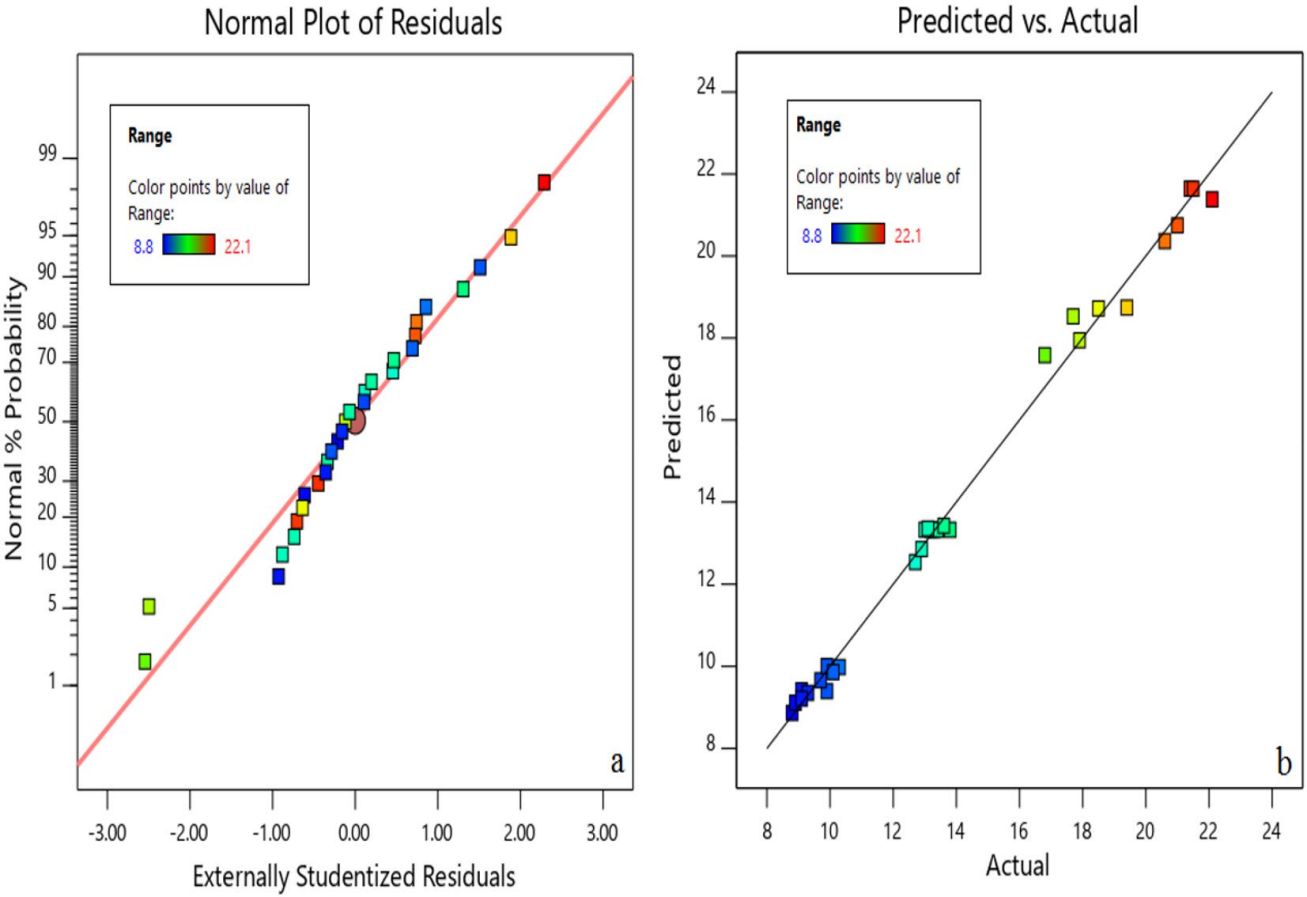
(**a**) Normal plot of residuals for CFCT cooling range (**b**) the acquired experimental values compared to the predicted cooling range values for TiO_2_ nanofluid.

Figure 7 illustrates the normal plot of residuals for the cooling range and the acquired experimental values compared to the predicted cooling range data for TiO_2_ nanofluid. It was observed that actual and predicted values have a good agreement in accordance with the obtained ${R}^{2}$ coefficient. Moreover, the residuals have acceptable proximity to the normal line. The color of the dots can detect different values of the actual cooling range. The same procedures were conducted, and similar results were obtained for MWCNTs nanofluid^28^.

**Figure 8:**
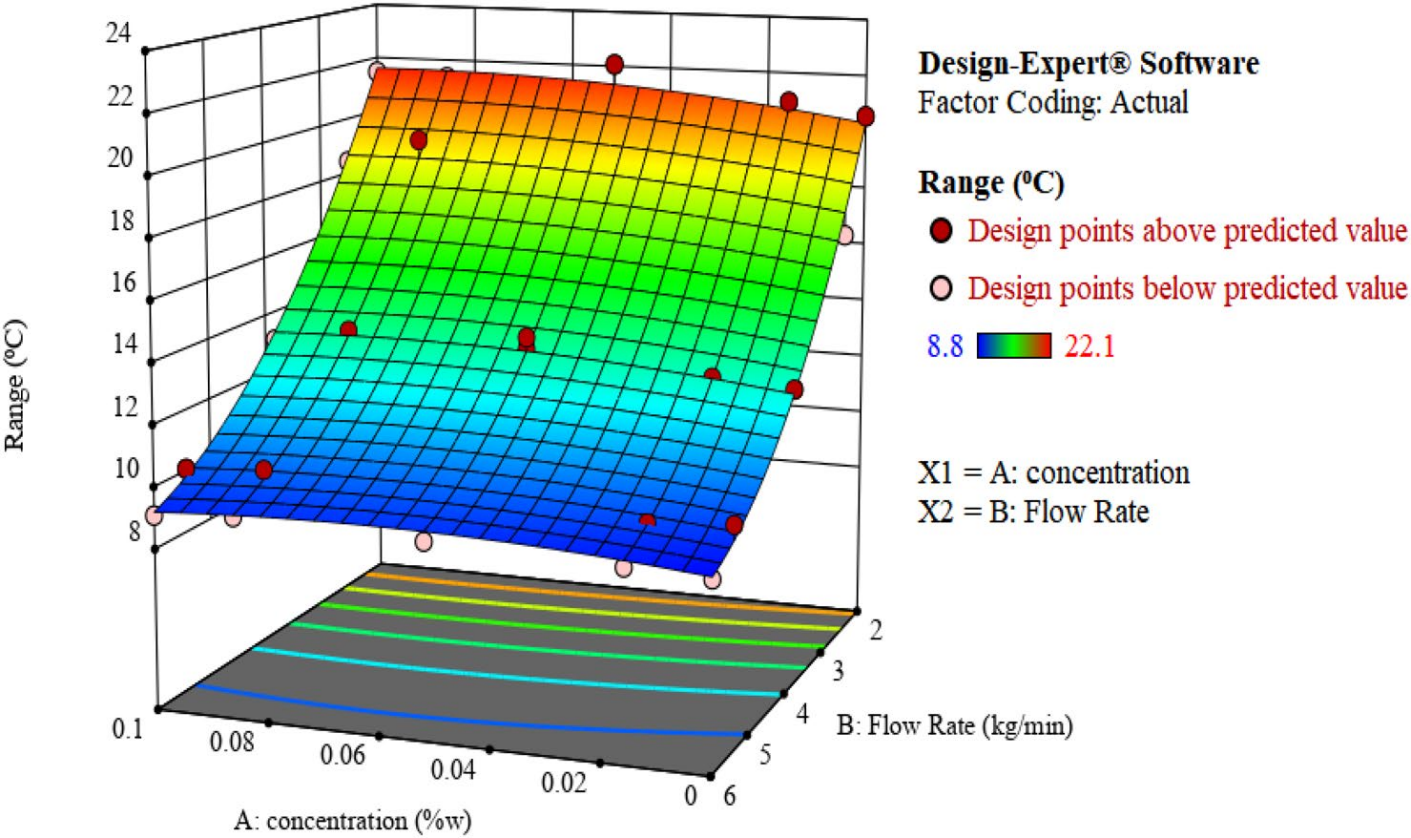
The 3D Surface and contour plot of the effect of independent variables on the cooling range using TiO_2_ nanofluid.

Figure 8 represents the effect of flow rate and nanofluid concentration on the cooling range of TiO_2_ nanofluid. As the flow rate of the TiO_2_ nanofluid increases, the temperature of outlet fluid decreases since the passing time of the fluid in the bed and the time for mass and heat transfer are reduced. The temperature of the inlet fluid decreases as the velocity of the circulating fluid increases due to the constant heating power; therefore, the cooling range of the tower is reduced. The same trend was seen for MWCNTs nanofluid. According to the obtained results considering two nanofluids, it can be concluded that increasing the flow rate led to lower performance of the cooling tower, and the cooling range is not dependent on the type of nanofluids^28^.

The effect of concentration on the cooling range can be analyzed by considering two states: low flow rates and high flow rates. Increasing the concentration of TiO_2_ nanofluid at low flow rates increased the cooling range. Based on the reported outcomes in the literature review, the addition of small amounts of nanoparticles to the base fluid could significantly improve the conductive heat transfer and, accordingly, the cooling range. At higher concentrations, in the range of 0.1 wt%, the trend becomes almost constant or slightly decreasing due to the agglomeration of nanoparticles and poor heat and mass transfer properties. It is observed that the cooling range in the range of 0.1 wt% is still higher than water, so the use of nanofluids, in any case, improves heat transfer and increases the cooling range of the tower.

The presence of the $AB$ term in the model equation becomes more highlighted at higher flow rates. The effect of flow rate on the cooling range is greater than the concentration at higher flow rates, demonstrating the interaction of flow rate and concentration on the response (cooling range). As a result, the impact of nanofluids on cooling tower performance is minimal at higher flow rates, and the lower the flow rate, the more significant the impact of nanofluids on cooling tower performance.

The effect of flow rate on cooling range compared to MWCNTs nanofluid concentration is known in all experiments. When the flow rate and concentration increase simultaneously, a kind of interactive competition is formed between these two factors affecting the cooling range, and the winner is the flow rate. According to the diagrams, the best flow rate for using MWCNTs nanofluid is also the lowest^28^.

**Figure 9:**
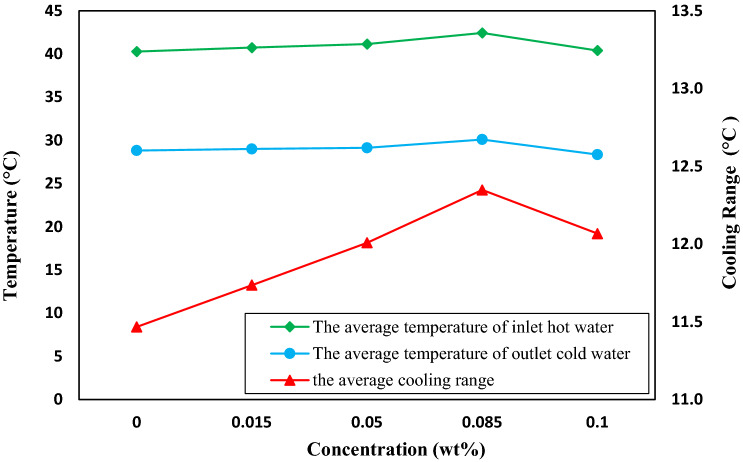
The average values of the inlet and outlet temperatures of the operating fluid and the cooling range of the five flow rates.

The average inlet and outlet temperatures of the operating fluid and cooling range of the five flow rates are presented in Fig. 9. The outlet cold water temperature is almost constant. However, increasing nanofluid concentration slightly increases the inlet temperature and the cooling range. Despite the equality of the received energy and the residence time of the fluid inside the heater, the nanofluid temperature rose more than water since the specific heat capacity of water in the operating temperature range of the tower was about six times that of TiO_2_, as the concentration of nanoparticles increased, the total heat capacity of the nanofluid decreased. As a result, a further rise in the temperature of the nanofluid relative to water with the same received energy could be related to the reduction in the specific heat capacity of the nanofluid compared to water.

**Figure 10:**
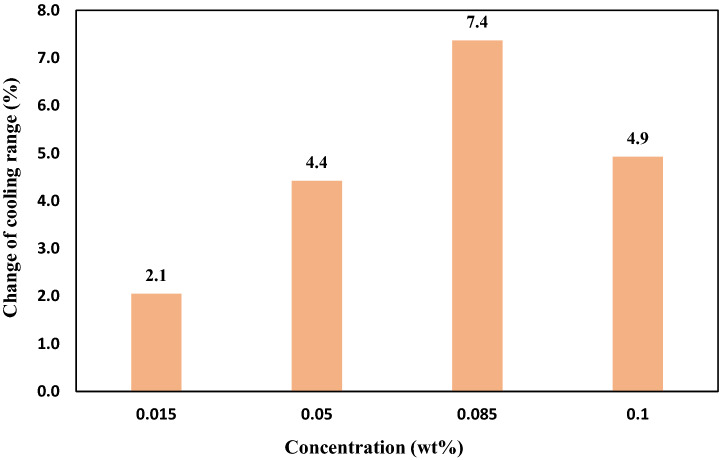
The effect of using nanofluid on the average cooling rates of five flow rates Using specified concentrations of TiO_2_ nanofluid relative to water.

Figure 10 exhibits the variation of the average cooling rates of five flow rates using specified concentrations of nanofluids. At the flow rate of 2–6 kg/min of circulating nanofluid, the optimum concentration of TiO_2_ nanofluid for the cooling process was 0.085 wt%. At this concentration, the average cooling range increased by 7.4%, while using MWCNTs nanofluid increased the cooling performance by 15.8%. Thus, using MWCNTs nanofluid had a significant enhancement impact on the cooling range than TiO_2_ nanofluid^28^.

### Changes in effectiveness as a function of concentration and flow rate

Firstly, the experiments were performed based on the listed data in Table 6, then using Eq. (2), the effectiveness of the tower was calculated and entered into the software. Table 9 lists the ANOVA table for tower effectiveness data of TiO_2_ nanofluid. The P-value of the Lack of Fit term is greater than 0.05 and is not significant, which indicates an acceptable agreement between the model and experimental results.

**Table 9:** The ANOVA table for effectiveness of TiO_2_ nanofluid.

Source	Sum of squares	df	Mean square	F-value	*p*-value
Model	1565.72	7	223.67	626.43	< 0.0001
A-concentration	1.66	1	1.66	4.64	0.0431
B-flow rate	60.10	1	60.10	168.32	< 0.0001
AB	1.05	1	1.05	2.94	0.1011
A^2^	3.4	1	3.40	9.51	0.0056
B^2^	11.52	1	11.53	32.26	< 0.0001
A^3^	1.11	1	1.11	3.11	0.0924
B^3^	6.41	1	6.41	17.96	0.0004
Residual	7.5	21	0.3571	–	–
Lack of fit	6.14	17	0.3614	1.07	0.5337
Pure error	1.35	4	0.3386	–	–
Cor total	1573.22	28	–	–	–

According to the significant terms of the model in the ANOVA table, the model equation is a modified cubic equation in which the insignificant terms were removed. To predict the effect of TiO_2_ nanofluid concentration and flow rate on the effectiveness, Eqs. (20) and (21) as the final equations in terms of actual and coded factors are presented herewith:

20 $$\mathrm{Effectiveness}=84.7-22.7\mathrm{C}-24.56\mathrm{L}+3.4\mathrm{CL}+1016.13{C}^{2}+4.73{L}^{2}-9113.73{C}^{3}-0.36{L}^{3}$$

21 $$\mathrm{Effectiveness}=40.08+1.2A-7.66B+0.34AB-0.88{A}^{2}+1.62{B}^{2}-1.14{A}^{3}-2.89{B}^{3}$$

To evaluate the validity of the proposed model, the descriptive statistics of the model for the effectiveness of CFCT using TiO_2_ nanofluid is given in Table 10.

**Table 10:** The descriptive statistics of the proposed model for effectiveness.

Statistical summary of the model	Statistical summary of data
${R}^{2}=$ 0.9952	Std. Dev.$=$ 0.5975
Adjusted ${R}^{2}=$ 0.9936	Mean $=$ 40.37
Predicted ${R}^{2}=$ 0.9907	C.V.%$=$ 1.48
Adeq Precision $=$ 71.322	–

The low value of the C.V. shows low data scatter. The R^2^ $=$ 0.9952 confirms the model’s ability to predict changes in the effectiveness of CFCT. The Adj-R^2^ $=$ 0.9936 indicates a 99.36% correlation between the model and experimental data. The model’s power in predicting points outside the defined levels is significant and had a value of 99.07%. Moreover, the difference between Pred-R^2^ and Adj-R^2^ is negligible, and Adeq Precision also has a substantial value of 71.322, showing the favorable conditions of the model for industry purposes.

For MWCNTs nanofluid, the insignificant terms were removed from the ANOVA table to increase the validity of the model. The P-value of the Lack of Fit term was more significant than 0.05 and negligible. The software presented a quadratic equation with ${R}^{2}=$ 0.9997 as the model equation, and the normal plot of residuals showed the good proximity of the residues to the normal line and in the diagram effectiveness values Predicted vs. Actual good agreement were observed between experimental and model data.

**Figure 11:**
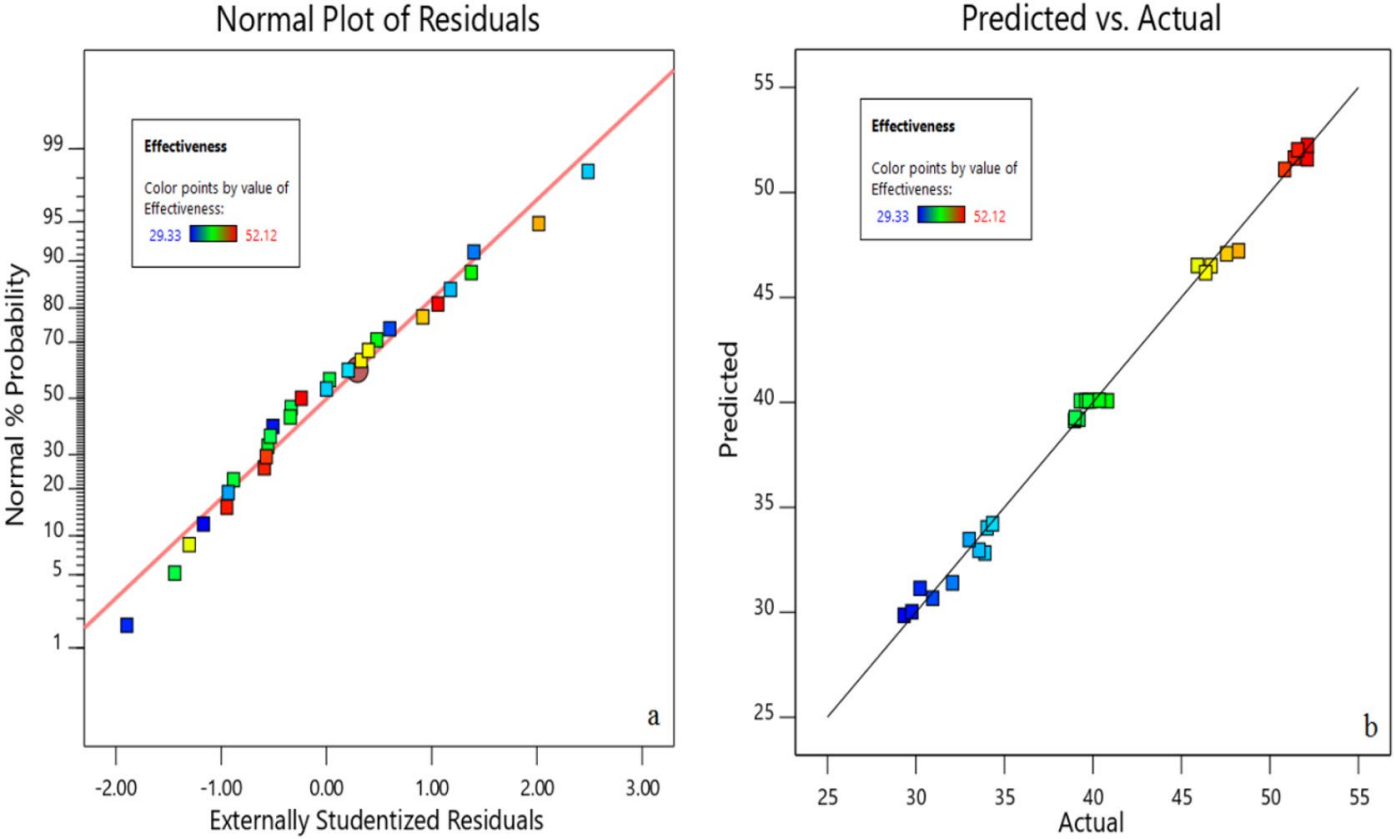
(**a**) Residual normal dispersion diagram for CFCT effectiveness (**b**) the comparison of the acquired experimental values with the predicted effectiveness values of TiO_2_ nanofluid.

Figure 11 represents the normal effectiveness plot of residuals and compares the acquired experimental values with the predicted effectiveness values of TiO_2_ nanofluid. The proximity of the data to the normal line and the conformity of the predicted data with the experimental data is acceptable.

**Figure 12:**
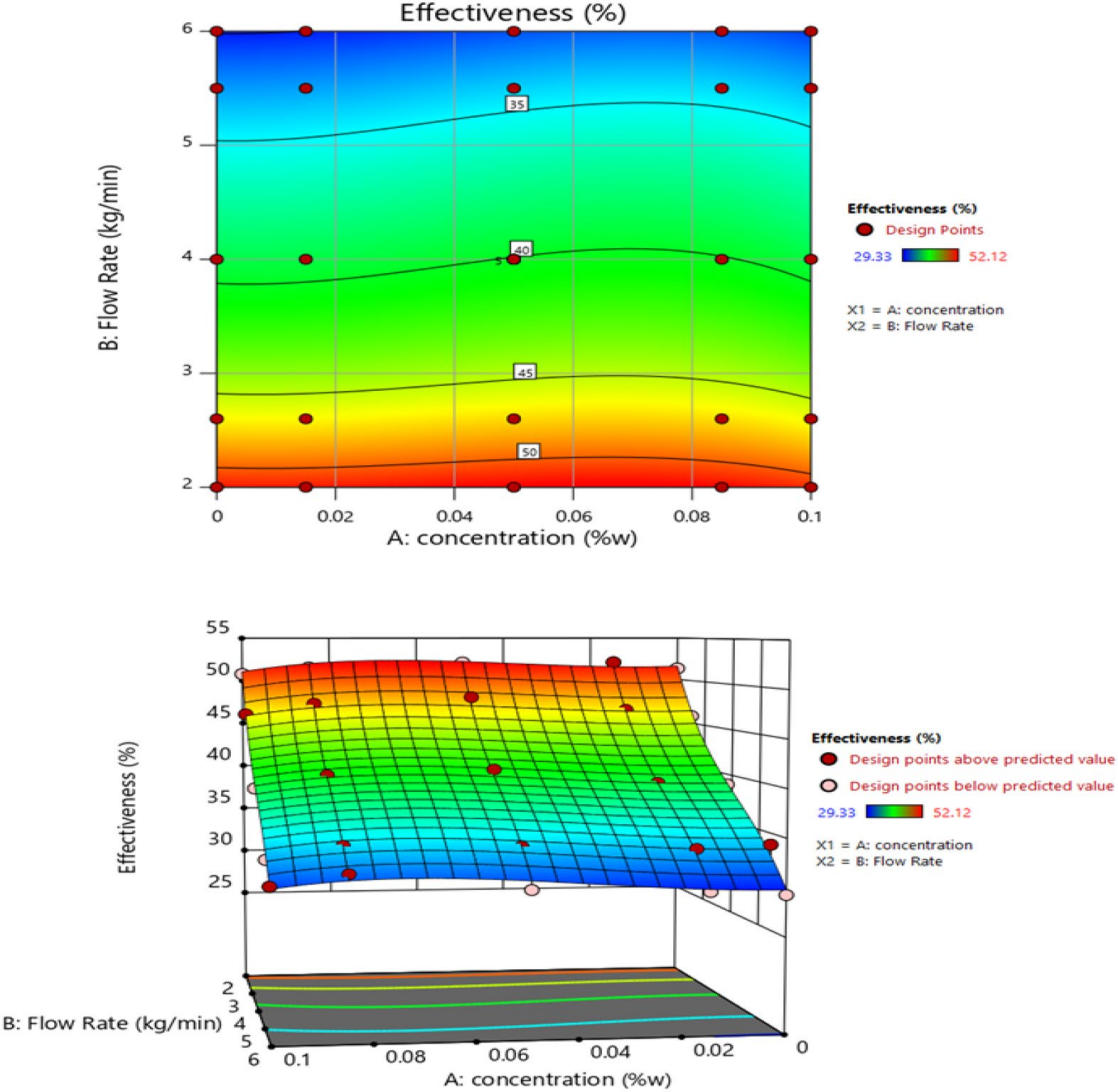
The effect of flow rate and concentration using TiO_2_ nanofluid on the effectiveness of CFCT, two-dimensional (contour) and three-dimensional diagrams.

The effect of flow rate and concentration of TiO_2_ nanofluid on the effectiveness of CFCT is shown in Fig. 12. The velocity of the circulating fluid increased with the increase of flow rate, leading to a decrease in the fluid's residence time inside the bed. Therefore, a heat and mass transfer time limit reduced the cooling range. On the other hand, the effectiveness of CFCT was influenced by its cooling range. Thus, the trend of changes in the tower's effectiveness was similar to the cooling range. It was observed that with increasing concentration up to about 0.08 wt%, the effectiveness initially increased and then decreased. The maximum effectiveness was in the concentration range of 0.08 wt%. Increasing the flow rate had a similar effect on the effectiveness of CFCT using MWCNTs nanofluids^28^.

**Figure 13:**
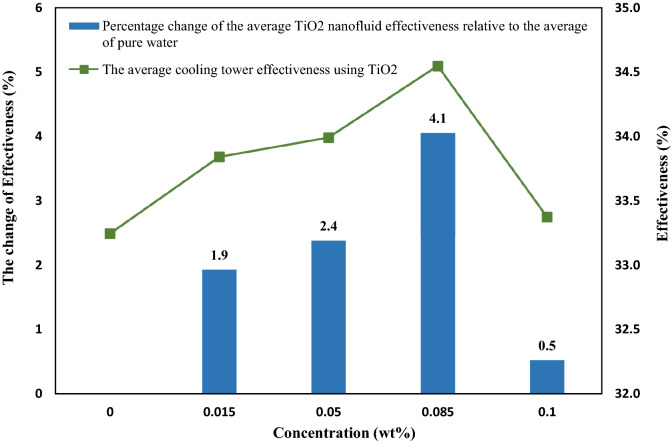
The average effectiveness and the percentage variation in the average effectiveness at specific concentrations in all the flow rates using nanofluids compared to pure water.

Figure 13 shows the average effectiveness of the five flow rates at specific concentrations. Also shown is the percent change in average effectiveness using nanofluids compared to pure water at specific concentrations. Nanofluid at 0.085 wt% showed the most remarkable improvement in effectiveness compared to pure water with a change of 4.1%, while the highest effectiveness using MWCNTs nanofluids at a similar concentration was 10.2%. Therefore, the use of MWCNTs shows better performance than TiO_2_ nanoparticles in improving the effectiveness^28^.

### Changes in Merkel number (transfer characteristic) as a function of concentration and flow rate

By performing the experiments considering data in Table 6 and Eq. (14), the Merkel number (transfer characteristic) of CFCT was obtained and entered into the software for verification**.** Tables 11 and 12 show the ANOVA data for the Merkel number of CFCT using MWCNTs and TiO_2_ nanofluids, respectively. The model's terms with a P-value $>$ 0.1 were removed from both tables, and the final values are provided in Tables 11 and 12. The Lack of Fit term is unimportant for both nanofluids, which revealed an acceptable agreement between the experimental and model results.

**Table 11:** The ANOVA table for Merkel number data of CFCT using MWCNTs nanofluid.

Source	Sum of squares	df	Mean square	F-value	*p*-value
Model	65.61	6	10.93	885.33	< 0.0001
A-concentration	0.1805	1	0.1805	14.61	0.0009
B-flow rate	2.10	1	2.10	170.13	< 0.0001
AB	0.1170	1	0.1170	9.47	0.0055
A^2^	0.4661	1	0.4661	37.74	< 0.0001
B^2^	0.0477	1	0.0477	3.87	0.0620
A^3^	0.4221	1	0.4221	34.18	< 0.0001
B^3^	0.2717	22	0.0124	_	–
Residual	0.2495	18	0.0139	2.49	0.5337
Lack of fit	0.0222	4	0.0056	–	–
Pure error	65.88	28	–	–	–
Cor total	65.61	6	10.93	885.33	< 0.0001

**Table 12:** The ANOVA table for Merkel number data of CFCT using TiO_2_ nanofluid.

Source	Sum of squares	df	Mean square	F-value	*p*-value
Model	70.10	7	10.01	2006.76	< 0.0001
A-concentration	0.0686	1	0.0686	13.75	0.0013
B-flow rate	2.61	1	2.61	523.69	< 0.0001
AB	0.0150	1	0.0150	3.00	0.0977
A^2^	0.0500	1	0.0500	10.02	0.0047
B^2^	0.0231	1	0.0231	4.64	0.0431
A^3^	0.0370	1	0.0370	7.41	0.0128
B^3^	0.3188	1	0.3188	63.88	< 0.0001
Residual	0.1048	21	0.0050	–	–
Lack of fit	0.0534	17	0.0031	0.2443	0.9833
Pure error	0.0514	4	0.0129	–	–
Cor total	70.21	28	–	–	–

According to the ANOVA table, the cubic model has the necessary conditions to fit the experimental data for both nanofluids. The P-value is less than 0.05 for the model and greater than this value for the Lack of Fit term, demonstrating that the model is significant, and the Lack of Fit data is not significantly related. The ${R}^{2}=$ 0.9959 for MWCNTs nanofluids and ${R}^{2}=$ 0.9985 for TiO_2_ nanofluids represent the high accuracy of the models presented for both nanofluids in describing response changes at surface points of independent variables (Tables 13 and 14).

**Table 13:** The descriptive statistics of the proposed model for Merkel number using MWCNTs nanofluid.

Statistical summary of the model	Statistical summary of data
${R}^{2}=$ 0.9959	Std. Dev.$=$ 0.1111
Adjusted ${R}^{2}=$ 0.9948	Mean $=$ 3.93
Predicted ${R}^{2}=$ 0.9927	C.V.%$=$ 2.83
Adeq Precision $=$ 87.7152	–

**Table 14:** The descriptive statistics of the proposed model for Merkel number using TiO_2_ nanofluid.

Statistical summary of the model	Statistical summary of data
${R}^{2}=$ 0.9985	Std. Dev.$=$ 0.0706
Adjusted ${R}^{2}=$ 0.9980	Mean $=$ 4.08
Predicted ${R}^{2}=$ 0.9974	C.V.%$=$ 1.73
Adeq Precision $=$ 124.8961	_

According to the significant terms of the model in the ANOVA table for MWCNTs and TiO_2_ nanofluids, the model equation for both nanofluids is the modified cubic equation, from which insignificant terms were removed. Equations (22) and (23) present the coded and realistic model equations for predicting the effect of concentration and flow rate of MWCNTs nanofluid, respectively. Also, Eqs. (24) and (25) are provided for TiO_2_ nanofluids.

22 $$\frac{1}{Me} = 4 - 0.4{\text{A }} + 1.43{\text{B }} + 0.16{\text{A}} - 0.33{\text{B}} + 0.24{\text{A}} + 0.74 {\text{B}}^{3}$$

23 $$\frac{1}{Me} = - 5.77 - 0.29{\text{C}} + 5.81{\text{L}} - 218.39{\text{C}} - 1.19 {\text{L}} + 1890.15{\text{C}} + 0.09{\text{L}}$$

24 $$\frac{1}{Me} = 4.04 - 0.13{\text{A}} + 1.6{\text{B}} + 0.04{\text{AB}} + 0.11{\text{A}}^{2} - 0.07{\text{B}}^{2} + 0.13{\text{AB}}^{2} + 0.64{\text{B}}^{3}$$

25 $$\frac{1}{Me} = 4.81 + 2.22{\text{C }} + 5.05{\text{L }} - 4.95{\text{CL }} + 42.58{\text{C}} - 1.02{\text{L}} + 0.67{\text{CL}} + 0.08{\text{L}}^{3}$$

According to the ANOVA table and the presented equations, the main difference between the two models is the terms that indicate the interaction of the final response between the two affecting factors, flow rate and concentration. In the TiO_2_ nanofluid model equation, flow rate and concentration interact with the final Merkel number due to $AB$ and ${AB}^{2}$ terms. In contrast, in the MWCNTs nanofluid model equation, these terms were removed from the final equation due to the large P-value.

**Figure 14:**
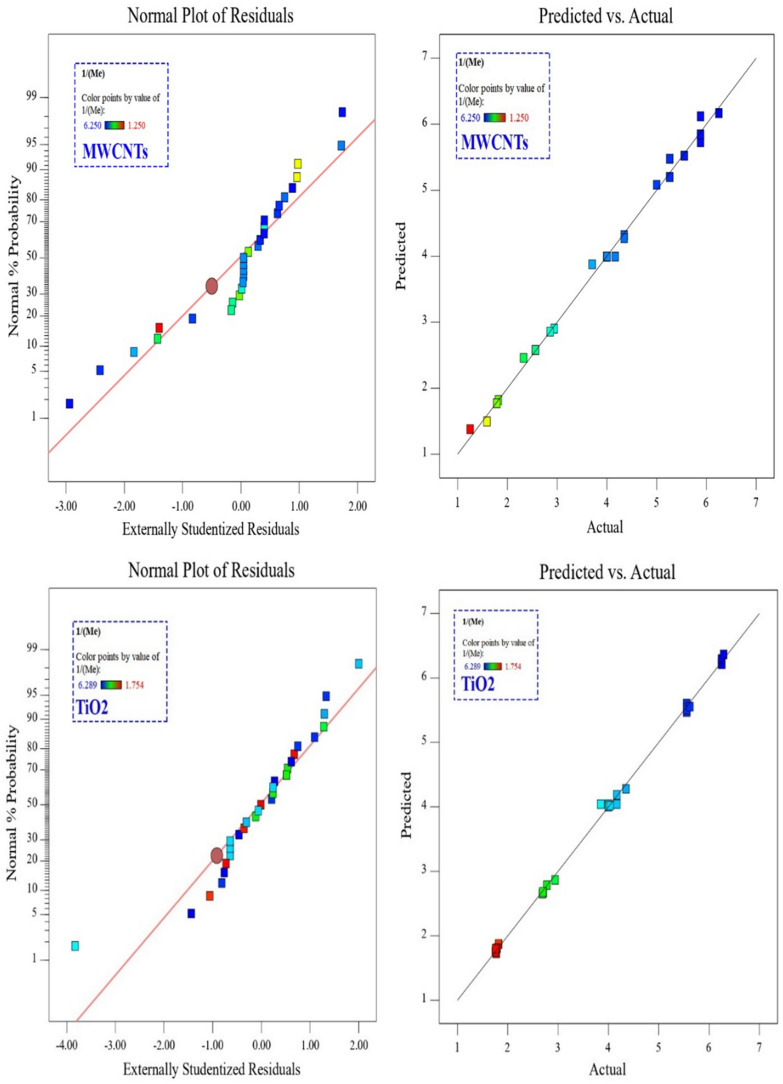
The normal residual diagrams of TiO_2_ and MWCNTs nanofluids comparing the expected Merkel number and experimental values.

Figure 14 shows the normal residual diagrams of TiO_2_ and MWCNTs nanofluids comparing the expected Merkel number and experimental values. The data for both MWCNTs and TiO_2_ nanofluids are near the normal line showing a good agreement between the acquired experimental values with the predicted values.

**Figure 15:**
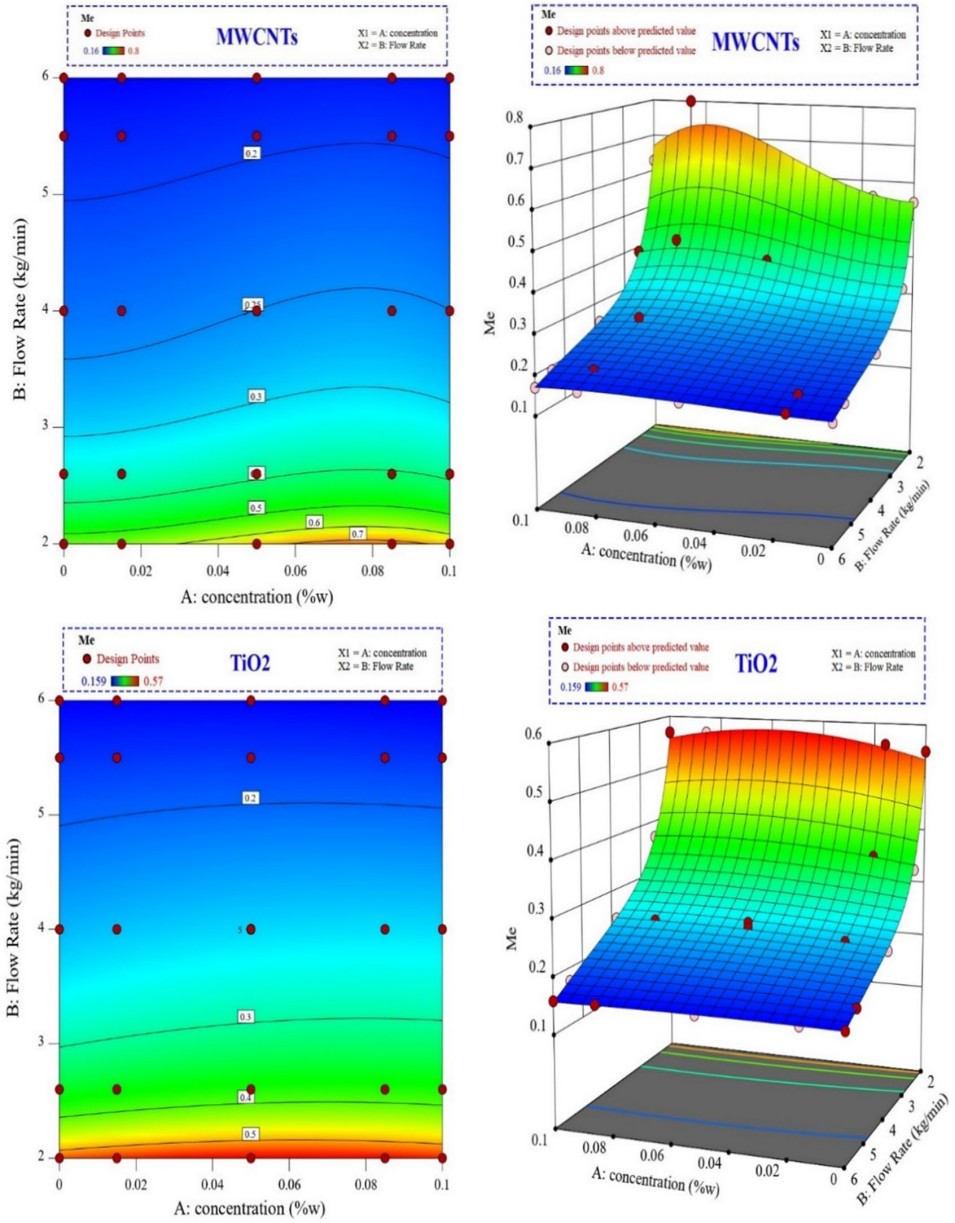
The influence of MWCNTs and TiO_2_ nanofluid concentration and flow rate on the Merkel number of CFCT in three-dimensional and contour diagrams.

Figure 15 depicts the influence of MWCNTs and TiO_2_ nanofluid concentration and flow rate on the Merkel number of CFCT in three-dimensional and contour diagrams. The Merkel number decreased as the flow rate of nanofluids increased, lessening the tower performance. Although raising the flow rate raised the Reynolds number and therefore the mass and heat transfer coefficient, the decrease in residence time and transfer time had a more significant effect, confirming the inverse relation between Merkel number and circulating fluid flow rate.

According to Fig. 15, the Merkel number was affected differently depending on the concentration of different flow rates. The change in Merkel number was more reliant on the concentration change at lower flow rates of both nanofluids. However, the influence of the concentration change on the Merkel number was minor at higher flow rates. The explanation for this is evident in Merkel's numerical relations. The mass transfer coefficient and total heat transfer coefficient increased as the concentration increased. As a result, the Merkel number increased.

Nevertheless, as previously explained, increasing the flow reduces the Merkel number. The increasing effect of concentration was higher than the decreasing effect of flow rates on the Merkel number at lower flow rates. However, flow rates highly affect the Merkel number at higher flow rates. The highest Merkel number for CFCT using MWCNTs and TiO_2_ nanofluids was reported at 0.08 and 0.06 wt%, respectively.

**Figure 16:**
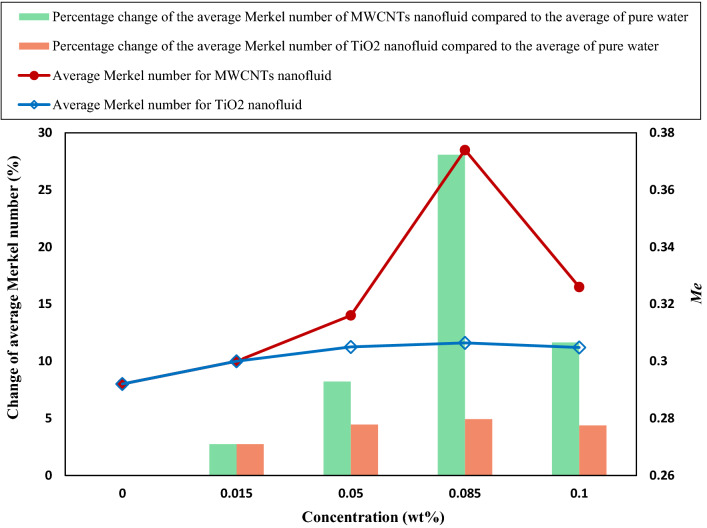
The average Merkel number of CFCT at diverse concentrations and five flow rates considering MWCNTs and TiO_2_ nanofluids.

Figure 16 presents the average Merkel number of CFCT at diverse concentrations and five specified flow rates considering MWCNTs and TiO_2_ nanofluids. It is observed that the Merkel number for MWCNTs and TiO_2_ nanofluids improved by about 28 and 5% compared to pure water, respectively. Furthermore, at almost all concentrations, the performance of MWCNTs nanofluids was better than TiO_2_ nanofluids.

### Optimization

The CFCT is optimal when the tower's cooling range, effectiveness, and Merkel number are at their highest possible values based on the process circumstances. Two independent variables, flow rate and concentration, must be set to maximize the abovementioned responses. The optimal values were obtained at low concentrations and high flow rates. Since the optimal condition was reported at lower concentrations, the cost-effectiveness of this process can be determined. Table 15 lists the software's optimization criteria for both nanofluids. The importance of each parameter in optimization was assigned a value between 1 and 5. For example, maximizing effectiveness is three times more important than minimizing nanofluid concentration. This decision was made owing to the importance of tower performance.

**Table 15:** The optimization conditions of MWCNTs and TiO_2_ nanofluids.

Parameter	Goal	Lower limit	Upper limit	Lower weight	Upper weight	Importance
A: concentration	Minimize	0	0.1	1	1	1
B: Flow rate	Maximize	2	6	1	1	1
Range	Maximize	8.8	25.2	1	1	2
Effectiveness	Maximize	31.14	56.47	1	1	3
*Me*	Maximize	0.16	0.8	1	1	5

The best conditions for each parameter using MWCNTs and TiO_2_ nanofluids selected by the program are listed in Table 16. Desirability, which has a value between zero and one, reflects how simple it is to achieve stated goals. The desirability of one implies that the stated goals are incredibly accessible and easy to attain. The program will likely give a large number of optimum spots. It is also more challenging to propose software to create targets to improve the value of optimization, and the ideal point reached.

**Table 16:** The optimal values proposed by software for each parameter for optimization of MWCNTs and TiO_2_ nanofluids.

Nanoparticle	Concentration (wt%)	Flow Rate (kg/min)	Range (°C)	Effectiveness (%)	*Me*	Desirability
MWCNTs	0.069	2.092	23.496	55.736	0.639	0.571
TiO_2_	0.033	2.116	20.551	50.796	0.510	0.650

The desirability of 0.571 presented for MWCNTs nanofluids shows that by adjusting the flow rate to 2.092 kg/min with a concentration of 0.069 wt% and a probability of 57.1%, the cooling range, effectiveness, and Merkel number of the tower will be 23.496, 55.736%, and 0.639, respectively. In addition, for TiO_2_ nanofluids, with a flow rate adjustment to 2.116 kg/min with a concentration of 0.033 wt% and a probability of 65%, the cooling range, efficiency, and Merkel number of the tower will be equal to 20.551, 50.796%, and 0.510, respectively.

The tests were repeated three times under optimal point circumstances to validate the optimal point, and the mean values are shown in Table 17. The reported values are inside the anticipated range and verify the optimal value's correctness. This illustrates the effectiveness of the response surface approach in optimizing cooling tower performance.

**Table 17:** Optimal point verification.

Nanoparticle	Response	Predicted mean	Predicted median	Std Dev	n	95% PI low	Data mean	95% PI high
MWCNTs	Range	23.4955	23.4955	0.0952851	3	23.3026	23.5667	23.6884
	Effectiveness	55.7358	55.7358	0.179157	3	55.402	55.7333	56.0696
	*Me*	0.639329	0.636149	0.045199	3	0.570809	0.658705	0.718382
TiO_2_	Range	20.553	20.5513	0.393571	3	19.9571	20.6667	21.1454
	Effectiveness	50.796	50.796	0.597549	3	49.8311	50.7667	51.761
	*Me*	0.510052	0.509392	0.0183544	3	0.482848	0.505025	0.539025

## Conclusion

In this study, the effect of concentration and flow rate of TiO_2_ nanofluid on the cooling characteristics of a cross-flow cooling tower was evaluated and compared with the new and former results of MWCNTs nanofluid. The measured properties, including effectiveness, Merkel number, and the cooling range, were compared using an experimental design by response surface methodology (RSM) based on the central composite design (CCD). The results revealed that nanofluids had a remarkable impact on substantially enhancing the cooling tower performance, especially at low flow rates. Furthermore, MWCNTs nanofluids had better efficacy than TiO_2_ nanofluids in improving the measured properties. To illustrate, 0.085 wt% MWCNTs nanofluid increased the Merkel number, efficacy, and cooling range by 28, 10.2, and 15.8 percent, respectively, whereas TiO_2_ nanofluid improved the mentioned properties by 5, 4.1, and 7.4 percent, respectively at the same content. The optimal setting of the system utilizing TiO_2_ nanofluid was suggested at a flow rate of 2.116 kg/min and a concentration of 0.033 wt%. The cooling range, effectiveness, and Merkel number under these conditions were 20.6, 50.8%, and 0.51, respectively. Despite the improved thermal performance, one of the major limitations of using nanofluids in heat transfer systems is their stability, which scientists have always been concerned with. It is suggested that more attention be paid to this aspect in future studies to make using nanofluids in cooling towers more feasible.

## Data Availability

All data generated or analysed during this study are included in this published article.

## List of symbols

a_fi_: Interfacial surface area between air and water per unit volume of fill zone(m^−^^1^)
A_fi_: Frontal area of fill perpendicular to air flow direction(m^2^)
C: Evaporative capacity rate ratio (kg s^−^^1^)
C_p_: Specific heat at constant pressure (j kg^−^^1^ k^−^^1^)
CFCT: Cross flow cooling tower
G: Mass velocity (kg m^−^^2^ s^−^^1^)
h_d_: Mass transfer coefficient (m s^−^^1^)
I: Specific enthalpy (j kg^−^^1^)
K: Air flow rate (kg s^−^^1^)
M: Mass flow rate (kg s^−^^1^)
M: Calculated quantity from the measurable parameter
Me: Merkel number
NTU: Number of transfer units
L_fi_: Fill length (m)
Q: Heat (W)
T: Temperature (°C)
T_R_: Cooling range (°C)
U: Maximum error
wt: Particle weight fraction (%)
y: Measurable parameters
X: Specific humidity of the air

## Greek symbols

ɛ: Effectiveness of the cooling tower
λ: Correction factor (pure number)

## Subscripts

*a*: Air
*bf*: Base fluid
*I*: Inlet
*Ma*: Air-vapor (per kg dry air)
*min*: Minimum
*max*: Maximum
*p*: Particle
*o*: Outlet
*s*: Saturated
*w*: Water
